# Compliance with the implementation of an ICU cluster-randomized trial assessing the benefits and potential harms of universal glove and gowning

**DOI:** 10.1186/cc11992

**Published:** 2013-03-19

**Authors:** D Kett, DJ Morgan, L Pineles, MJ Zervos, LS Munoz-Price, AD Harris, BUGG Investigators

**Affiliations:** 1 University of Miami/Jackson Memorial Hospital, Miami, FL, USA; 2 University of Maryland/VA Maryland Healthcare System, Baltimore, MD, USA; 3 Henry Ford Health System, Detroit, MI, USA

## Introduction

The Benefits of Universal Glove and Gowning (BUGG) study is a cluster-randomized trial to evaluate the use of wearing gloves and gowns for all patient contact in the ICU. The primary outcome is VRE and MRSA acquisitions; secondary outcomes include frequency of healthcare worker visits, infection rates, hand hygiene compliance and adverse events.

## Methods

We enrolled 20 ICUs in 15 states. ICUs collected nasal and perianal swabs on all patients at admission and discharge/transfer. After a 3-month baseline period, 10 units were randomized to the intervention arm and required to wear gloves and gowns for all patient contact. An intervention toolkit was created based on site feedback and compliance reports. Swab collection compliance was fed back and discussed during site conference calls on a weekly basis. Site coordinators monitored compliance with gloves and gowns, hand hygiene and frequency of HCW visits and reviewed patient charts for adverse events.

## Results

**Figure 1:**
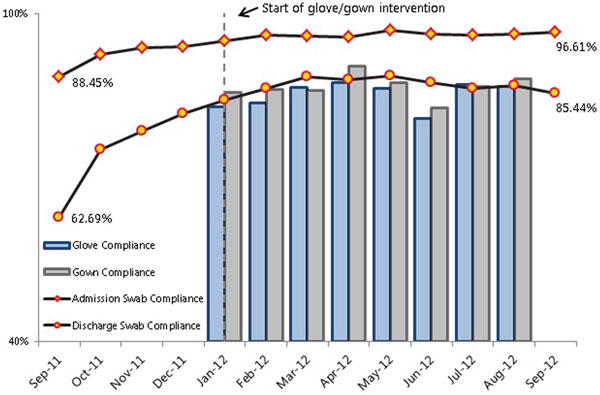
**Swab collection and gown/glove compliance**.

During the 12-month study period, 100,210 swabs were collected. After the baseline period, we were able to achieve and maintain swab compliance rates between 85 and 97%. Monthly discharge compliance increased by 21% by the beginning of the intervention period (Figure 1). Observers found 86% compliance with universal glove and gowning over 1,242 30-minute observation periods (Figure 1). Ninety charts at each site were reviewed for adverse events.

## Conclusion

Over a diverse group of US hospitals, we achieved high compliance with surveillance cultures and implementing universal gloving and gowning was achieved quickly with high compliance.

